# The relationship between perihematomal edema and hematoma expansion in acute spontaneous intracerebral hemorrhage: an exploratory radiomics analysis study

**DOI:** 10.3389/fnins.2024.1394795

**Published:** 2024-04-30

**Authors:** Zhiming Zhou, Xiaojia Wu, Yuanyuan Chen, Yuanxin Tan, Yu Zhou, Tianxing Huang, Hongli Zhou, Qi Lai, Dajing Guo

**Affiliations:** ^1^Department of Radiology, Second Affiliated Hospital of Chongqing Medical University, Chongqing, China; ^2^Chongqing Medical Imaging Artificial Intelligence Lab, Chongqing, China; ^3^Department of Radiology, Fifth People's Hospital of Chongqing, Chongqing, China; ^4^Department of Radiology, Nanchong Central Hospital, Nanchong, Sichuan, China

**Keywords:** Radiomics, computed tomography, perihematomal edema, intracerebral hemorrhage, hematoma expansion

## Abstract

**Background:**

The relationship between early perihematomal edema (PHE) and hematoma expansion (HE) is unclear. We investigated this relationship in patients with acute spontaneous intracerebral hemorrhage (ICH), using radiomics.

**Methods:**

In this multicenter retrospective study, we analyzed 490 patients with spontaneous ICH who underwent non-contrast computed tomography within 6 h of symptom onset, with follow-up imaging at 24 h. We performed HE and PHE image segmentation, and feature extraction and selection to identify HE-associated optimal radiomics features. We calculated radiomics scores of hematoma (Radscores_HEA) and PHE (Radscores_PHE) and constructed a combined model (Radscore_HEA_PHE). Relationships of the PHE radiomics features or Radscores_PHE with clinical variables, hematoma imaging signs, Radscores_HEA, and HE were assessed by univariate, correlation, and multivariate analyses. We compared predictive performances in the training (*n* = 296) and validation (*n* = 194) cohorts.

**Results:**

Shape_VoxelVolume and Shape_MinorAxisLength of PHE were identified as optimal radiomics features associated with HE. Radscore_PHE (odds ratio = 1.039, *p* = 0.032) was an independent HE risk factor after adjusting for the ICH onset time, Glasgow Coma Scale score, baseline hematoma volume, hematoma shape, hematoma density, midline shift, and Radscore_HEA. The areas under the receiver operating characteristic curve of Radscore_PHE in the training and validation cohorts were 0.808 and 0.739, respectively. After incorporating Radscore_PHE, the integrated discrimination improvements of Radscore_HEA_PHE in the training and validation cohorts were 0.009 (*p* = 0.086) and −0.011 (*p* < 0.001), respectively.

**Conclusion:**

Radscore_PHE, based on Shape_VoxelVolume and Shape_MinorAxisLength of PHE, independently predicts HE, while Radscore_PHE did not add significant incremental value to Radscore_HEA.

## Introduction

Intracerebral hemorrhage (ICH) is a major cause of global mortality (An et al., 2017). Hematoma expansion (HE), defined as an increase in the volume of the initial ICH over time, has garnered considerable attention due to its association with neurological deterioration and poor prognosis (Steiner and Bosel, 2010; Brouwers and Greenberg, 2013). Perihematomal edema (PHE) is typically induced within the initial 3 h after ICH, with an approximately 75% increase in absolute volume observed within the first 24 h (Gebel et al., 2002). However, the relationship among PHE, intracerebral hematoma, and HE in patients with acute spontaneous ICH is not well established.

Computed tomography (CT) is the most widely used method for evaluating ICH. Extensive research has identified the initial hematoma volume as an independent predictor of HE (Morotti et al., 2019a; Morotti and Arba, 2020). However, the precise relationship between PHE and hematoma volume remains controversial. While some studies have suggested a potential correlation between the PHE volume and baseline hematoma volume within the initial hours post-onset (Arima et al., 2009; Staykov et al., 2011; Venkatasubramanian et al., 2011), conflicting findings exist, with certain investigations failing to establish a definitive association between these two features (Gebel et al., 2002). Notably, few studies have explored the interplay between PHE volume and HE, with existing studies yielding conflicting findings regarding the association between absolute PHE volume and HE, as compared to relative PHE volume, which showed no significant correlation with HE (Gebel et al., 2002; Rodriguez-Luna et al., 2016).

In addition to hematoma volume, specific radiological markers indicative of heterogeneous density or irregular morphology have emerged as predictors of HE (Wada et al., 2007; Barras et al., 2009; Morotti et al., 2019a). Furthermore, one study noted that patients presenting with spot signs within the hematoma exhibited a larger baseline PHE volume (Rodriguez-Luna et al., 2016). Radiomics analysis can be used to evaluate lesion characteristics by extracting high-dimensional features from medical images. Compared to traditional visual assessments, radiomics analysis offers comprehensive, objective, and quantitative evaluations. A recent meta-analysis has shown the efficacy of radiomics features extracted from CT images in characterizing the heterogeneity of ICH and predicting HE (Jiang et al., 2022). Additionally, emerging evidence has indicated a strong correlation between the CT-based radiomics features of PHE and functional prognosis (Huang et al., 2022, 2023). However, to the best of our knowledge, research investigating the predictive value of CT-based radiomics features of PHE in HE has been limited to date.

We hypothesized that the radiomics features of PHE derived from noncontrast CT (NCCT) may be associated with HE. The aims of this study were twofold: first, to determine the potential correlation between radiomics features or radiomics score (Radscore) of PHE and HE, and second, to evaluate whether the addition of the PHE Radscore (Radscore_PHE) to the Radscore of hematoma (Radscore_HEA) improves the predictive capability for HE.

## Materials and methods

**Figure 1:**
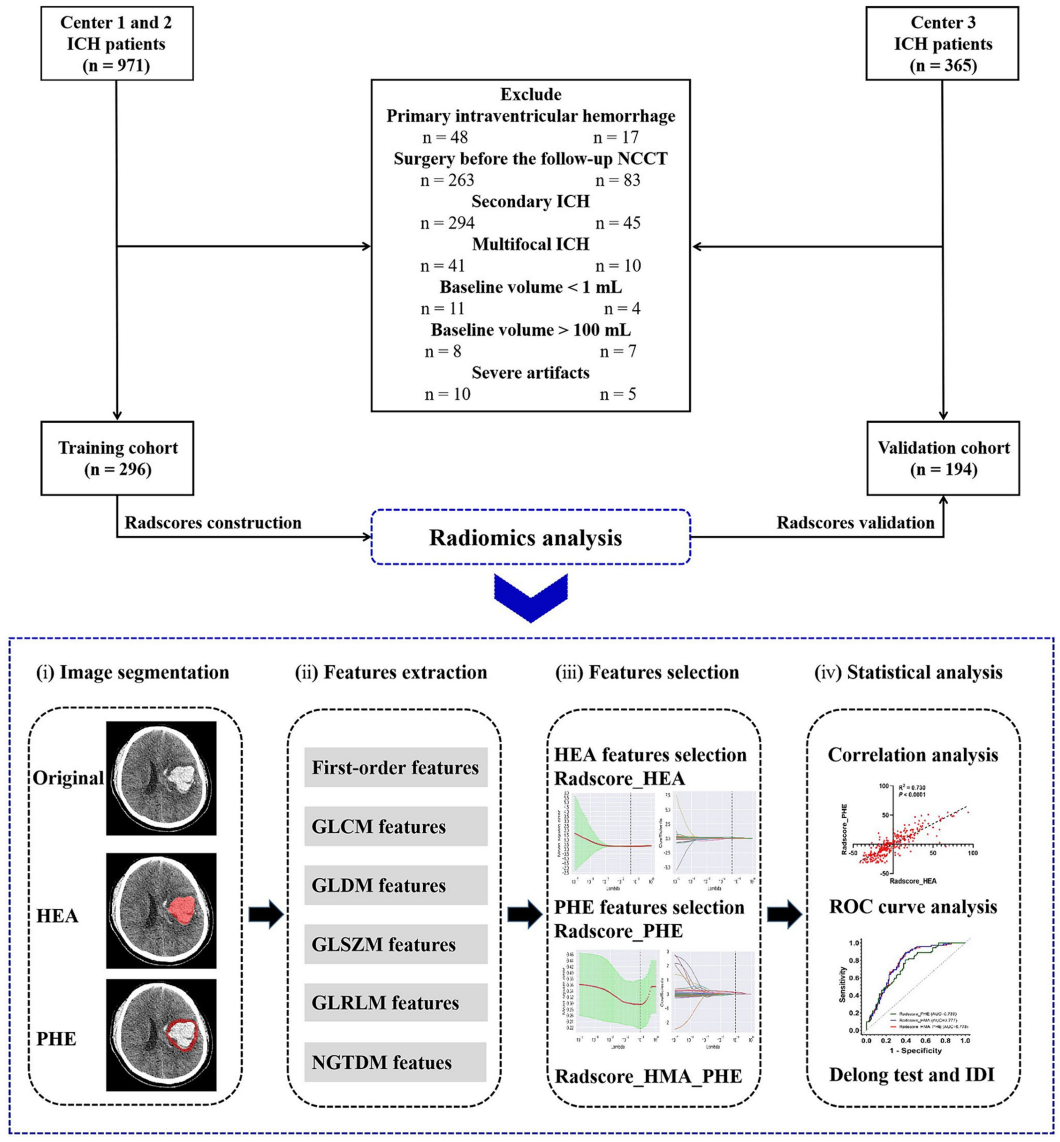
Flow chart of this study.

This study was approved by the institutional review board of our hospital (decision number [2019] 19). The requirement for obtaining informed consent was waived due to the retrospective nature of the study. A flowchart of this study is illustrated in Figure 1.

### Study population

We retrospectively reviewed 1,336 patients diagnosed with ICH at three medical centers in China between 2014 and 2021. Patients from centers 1 and 2 in Chongqing served as the training cohort, whereas those from center 3 in Sichuan served as the validation cohort.

The inclusion criteria were age > 18 years, available baseline NCCT obtained within 6 h of ICH onset, and available follow-up NCCT obtained within 24 h of baseline NCCT. The exclusion criteria were as follows: primary intraventricular hemorrhage (IVH) or secondary ICH; baseline hematoma volume > 100 mL or < 1 mL; multifocal ICH; surgery before follow-up NCCT; and severe image artifacts.

### Clinical data collection

The clinical data of patients at admission were collected from electronic medical records, including sex, age, hypertension history, diabetes history, warfarin treatment history, antiplatelet treatment history, time from ICH onset (Onset_time), Glasgow Coma Scale (GCS) score, and blood pressure.

### Image acquisition and preprocessing

Head NCCT data were acquired using three multi-detector CT scanners with the standard scanning protocol of a tube voltage of 120 kV, smart mAs, slice thicknesses of 1 mm or 1.25 mm, a matrix size of 512 × 512, field of view of 25 cm, and gantry rotation of 0.4–0.6 s. Scanning range was performed from the skull base to the cranium.

Image preprocessing was performed to normalize images from different CT scanners, including image resampling: every NCCT image slice from the raw data was resampled to a unified pixel dimension size of 1.0 × 1.0 × 1.0 mm^3^; gray-level discretization processing: image intensity of every NCCT image was normalized by the gray-level discretization method with a fixed number of 256 bins; standard head window setting: NCCT images were viewed in a fixed head with window level of 50 Hounsfield unit (Hu) and window width of 110 Hu.

### Image analysis and segmentation

With reference to previous literature (Barras et al., 2009; Yang et al., 2018; Zhou et al., 2021), imaging characteristics, including hematoma volume, hematoma location (lobar, deep, brainstem, or cerebellum), hematoma shape (regular or irregular), hematoma density (homogeneity or heterogeneity), midline shift, IVH, and the occurrence of HE, were independently assessed and recorded by a trained neuroradiologist (with 7 years of work experience), blinded to patients’ clinical status. The baseline and follow-up hematoma volumes were automatically calculated using artificial intelligence software (StrokeDoc).^1^ HE was defined as either a proportional increase of >33% or an absolute increase of >6 mL in the volume of the two hematomas (Demchuk et al., 2012).

The volumes of interest (VOIs) of the baseline hematoma and PHE were manually segmented by three trained neuroradiologists (with 5, 3, and 3 years of working experience, respectively) using the open source software (ITK-SNAP)^2^ with discussion to consensus where necessary. Perihematomal hypodensity encircling the hematoma in ICH cases is typically identified as PHE. The demarcation of the edema borders can be facilitated by referencing the CT attenuation values of the unaffected brain parenchyma on either the ipsilateral or contralateral side. When PHE was undetectable, PHE characteristic values of zero were assigned.

### Feature extraction and selection

For each VOI, the radiomics features of the hematoma and PHE were extracted using Python software.^3^ To evaluate the reproducibility of the radiomics features of the hematoma and PHE, we performed reproducibility analyses and interclass correlation coefficients (ICC) in a randomized sample of 50 patients obtained from the training cohort, as detailed in the Supplementary material.

Before feature selection, we used Python software to perform harmonization in the feature domain, with exclusion of features with zero variance, replacement of missing values of PHE features with zero values, and standardization of feature values. We then used the univariate analysis and elastic net regression to select the radiomics features of hematoma and PHE most associated with HE, respectively.

### Radscores construction and validation

In the training cohort, the Radscores of hematoma (Radscore_HEA) and PHE (Radscore_PHE) were calculated using the values of the optimal radiomics features multiplied by the corresponding coefficients in the elastic net regression. The two Radscores were then combined using logistic regression to construct a combined model (Radscore_HEA_PHE) for HE. In the validation cohort, these three radscores were validated to predict HE.

### Statistical analysis

Univariate analysis including the chi-square test, Fisher’s exact test, Student’s *t*-test, and Mann–Whitney *U* test was used for comparison between groups. Multivariate analysis was used to determine the independent risk factors for HE. Spearman’s correlation analysis was used to assess the correlations between different variables. Receiver operating characteristic (ROC) curve analysis was used to assess the discriminatory power of Radscores. The area under the ROC curve (AUC) was calculated. The Delong test was used to compare the ROC curves between different Radscores, and integrated discrimination improvement (IDI) was used to test the incremental predictive value of Radscore_PHE after adding it to Radscore_HEA. Statistical analysis was performed with SPSS (IBM Inc., Armonk, NY, United States), Python, or R software.^4^ A two-sided *p* value <0.05 was considered statistically significant.

## Results

### Patient characteristics

In total, 490 patients with spontaneous ICH were enrolled, including 296 from Chongqing (training cohort) and 194 from Sichuan (validation cohort), (Figure 1). The characteristics of the patients in the two cohorts are summarized in Table 1. Of these, 177 (36.1%) experienced HE. Patient characteristics did not differ significantly between the training and validation cohorts, except for the baseline PHE volume (*p* = 0.003).

**Table 1:** Patient characteristics in the training and validation cohorts.

Variables	Training cohort (*n* = 236)	Validation cohort (*n* = 194)	*p*
Sex			0.435
Male	176 (59.5)	123 (63.4)	
Female	120 (40.5)	71 (40.5)	
Age, y	60.80 ± 13.60	60.85 ± 14.87	0.970
Hypertension history	207 (69.9)	122 (62.9)	0.127
Diabetes history	245 (82.8)	167 (86.1)	0.393
Warfarin treatment history	10 (3.4)	7 (3.6)	1.000
Antiplatelet treatment history	24 (8.1)	19 (9.8)	0.630
Onset_time, h	2.00 [1.00, 5.00]	3.00 [2.00, 4.00]	0.187
GCS score	13.00 [10.00, 15.00]	13.00 [10.00, 14.00]	0.056
Systolic blood pressure, mmHg	173.73 ± 29.72	169.77 ± 28.79	0.145
Diastolic blood pressure, mmHg	99.94 ± 17.85	98.76 ± 17.57	0.470
Midline shift	85 (28.7)	63 (32.5)	0.432
IVH	89 (30.1)	60 (30.9)	0.919
Location			0.128
Lobar	43 (14.5)	38 (19.6)	
Deep	228 (77.0)	141 (72.7)	
Brain stem	16 (5.4)	5 (2.6)	
Cerebellum	9 (3.0)	10 (5.2)	
Irregular shape	192 (64.9)	132 (68.0)	0.529
Heterogeneous density	96 (32.4)	66 (34.0)	0.789
Baseline hematoma volume, mL	11.38 [5.16, 22.47]	12.52 [5.83, 22.88]	0.494
Baseline PHE volume, mL	11.36 [5.41, 20.00]	14.49 [8.00, 23.69]	0.003
Follow-up hematoma volume, mL	12.65 [5.55, 28.30]	16.27 [6.22, 29.57]	0.175
HE	105 (35.5)	72 (37.1)	0.784
Radscore_HEA	0 ± 24.12	0 ± 24.99	> 0.999
Radscore_PHE	0 ± 19.88	0 ± 19.80	> 0.999

Data are noted as mean and standard deviation, median and interquartile ranges or numbers and percentages in parenthesis. Onset_time, time from ICH onset; GCS, Glasgow coma scale; IVH, intraventricular hemorrhage; PHE, perihematomal edema; HE, hematoma expansion; HEA, hematoma.

Significant differences in Onset_time, GCS score, midline shift, hematoma shape, hematoma density, baseline hematoma volume, baseline PHE volume, and follow-up hematoma volume, were found between the HE group and non-HE group in the training cohort (Table 2). The Onset_time and GCS score were used to construct a clinical score by logistic regression.

**Table 2:** Univariable analysis for HE in the training cohort.

Variables	Non-HE group (*n* = 191)	HE group (*n* = 105)	*p*
Sex			0.792
Male	112 (58.6)	64 (61.0)	
Female	79 (41.4)	41 (39.0)	
Age, y	61.63 ± 13.15	59.29 ± 14.31	0.156
Hypertension history	131 (68.6)	76 (72.4)	0.583
Diabetes history	34 (17.8)	17 (16.2)	0.849
Warfarin treatment history	8 (4.2)	2 (1.9)	0.481
Antiplatelet treatment history	18 (9.4)	6 (5.7)	0.370
Onset_time, h	3.00 [2.00, 5.00]	2.00 [1.00, 3.00]	< 0.001
GCS score	14.00 [11.00, 15.00]	11.00 [10.00, 13.00]	< 0.001
Systolic blood pressure, mmHg	172.04 ± 28.32	176.80 ± 32.02	0.188
Diastolic blood pressure, mmHg	99.28 ± 16.62	101.14 ± 19.92	0.392
Midline shift	34 (17.8)	51 (48.6)	< 0.001
IVH	53 (27.7)	36 (34.3)	0.298
Location			0.059
Lobar	22 (11.5)	21 (20.0)	
Deep	148 (77.5)	80 (76.2)	
Brain stem	13 (6.8)	3 (2.9)	
Cerebellum	8 (4.2)	1 (1.0)	
Irregular shape	113 (59.2)	79 (75.2)	0.008
Heterogeneous density	42 (22.0)	54 (51.4)	< 0.001
Baseline hematoma volume, mL	8.60 [3.90, 14.67]	21.94 [11.34, 41.81]	< 0.001
Baseline PHE volume, mL	7.86 [4.36, 13.54]	19.45 [12.38, 30.31]	< 0.001
Follow-up hematoma volume, mL	8.40 [3.48, 14.77]	31.86 [19.12, 52.48]	< 0.001
Radscore_HEA	−10.30 ± 15.18	18.73 ± 26.09	< 0.001
Radscore_PHE	7.81 ± 14.58	14.20 ± 20.42	< 0.001

Data are noted as mean and standard deviation, median and interquartile ranges or numbers and percentages in parenthesis. HE, hematoma expansion; Onset_time, time from ICH onset; GCS, Glasgow coma scale; IVH, intraventricular hemorrhage; PHE, perihematomal edema; HEA, hematoma.

### Radiomics analysis

For each VOI, 107 radiomics features were extracted, as detailed in the Supplementary material.

Intra-observer reproducibility showed that 103 (96.3%) radiomics features of hematoma and 100 (93.5%) radiomics features of PHE had ICCs >0.8. Inter-observer reproducibility evaluation showed that 101 (94.4%) radiomics features of hematoma and 97 (90.7%) radiomics features of PHE had ICCs >0.8.

**Figure 2:**
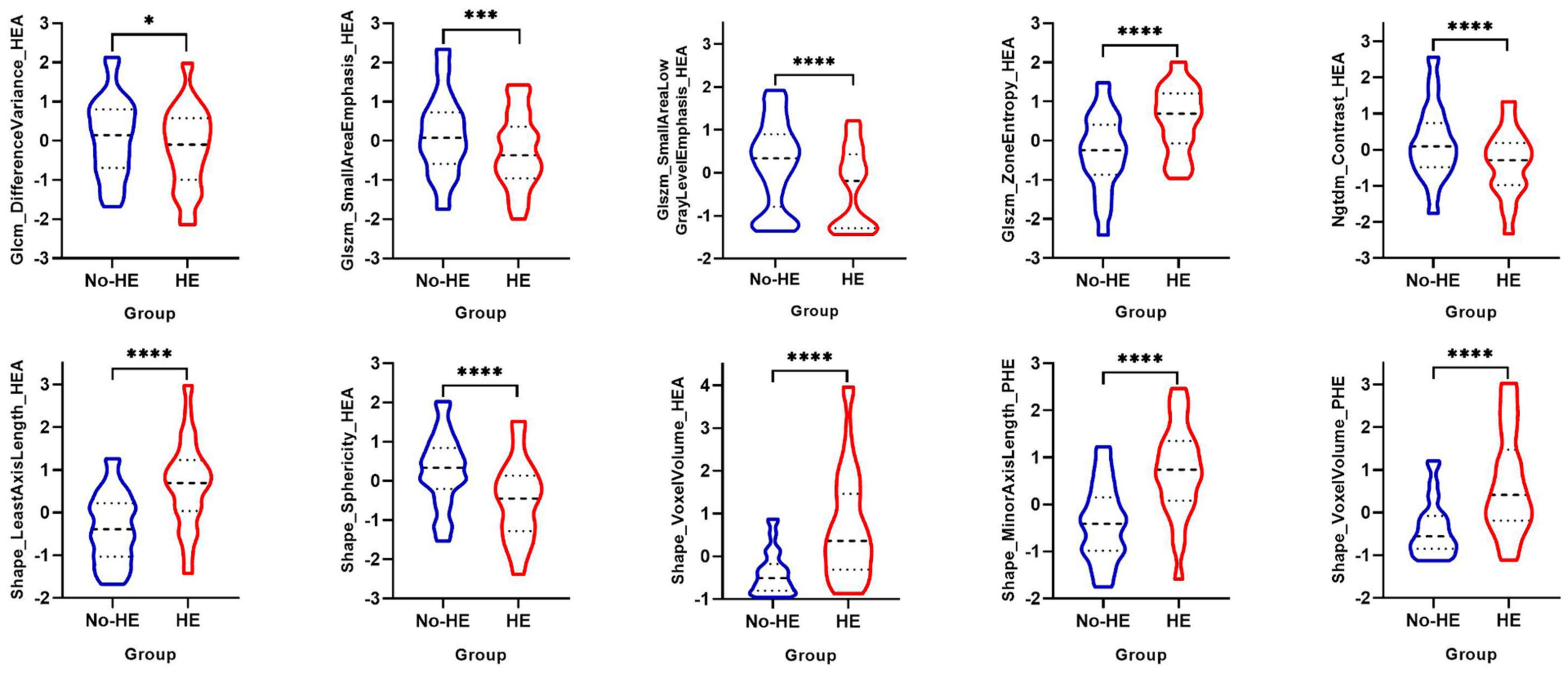
Univariable analysis of optimal radiomics features of hematoma and PHE between the HE group and the non-HE group in the training cohort. PHE, perihematomal edema; HE, hematoma expansion. *p < 0.05; ***p < 0.001; ****p < 0.0001.

The process of feature selection is detailed in the Supplementary material. After feature selection, eight radiomics features of hematoma and two radiomics features of PHE were identified as optimal features associated with HE (Figure S1 in Supplementary material). In the training cohort, there were significant differences in Glcm_DifferenceVariance_HEA (*p* = 0.02), Glszm_SmallAreaEmphasis_HEA (*p* < 0.001), Glszm_SmallAreaLowGrayLevelEmphasis_HEA (*p* < 0.001), Glszm_ZoneEntropy_HEA (*p* < 0.001), Ngtdm_Contrast_HEA (*p* < 0.001), Shape_LeastAxisLength_HEA (*p* < 0.001), Shape_Sphericity_HEA (*p* < 0.001), Shape_VoxelVolume_HEA (*p* < 0.001), Shape_MinorAxisLength_PHE (*p* < 0.001), Shape_VoxelVolume_PHE (*p* < 0.001) between the HE group and the non-HE group (Figure 2).

There were no significant differences in the Radscore_HEA (*p* > 0.999) or Radscore_PHE (*p* > 0.999) between the training and validation cohorts (Table 1). In the training cohort, both Radscore_HEA (*p* < 0.001) and Radscore_PHE (*p* < 0.001) were significantly higher in the HE group than in the non-HE group (Table 2).

### Analysis of PHE radiomics features and Radscore_HEA

**Figure 3:**
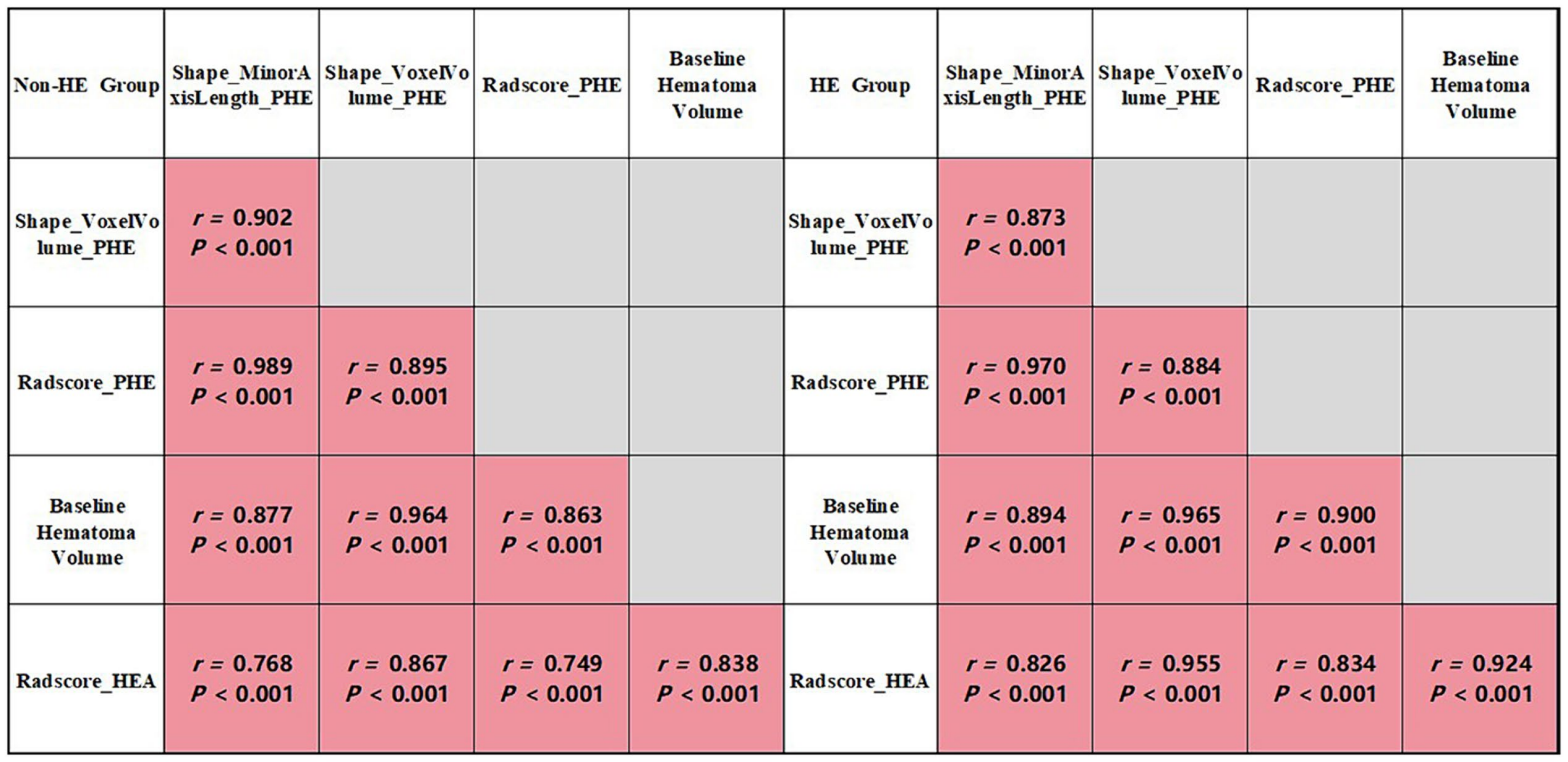
Correlation analysis of group-wise quantification in the training cohort.

The results of the univariate and Spearman correlation analyses for optimal PHE radiomics features and Radscore_HEA in the training cohort are summarized in Table 3 and Figure 3. In the training cohort, the Shape_MinorAxisLength_PHE, Shape_VoxelVolume_PHE, and Radscore_HEA of the midline shift, heterogeneous, and irregularly shaped groups were significantly higher than those of the non-midline shift, homogeneous, and regularly shaped groups (all *p* < 0.001), and these three variables were significantly associated with the GCS score, Onset_time, baseline hematoma volume, and Radscore_HEA (all *p* < 0.001). In addition, the Shape_MinorAxisLength_PHE, Shape_VoxelVolume_PHE, and Radscore_PHE of the HE group were significantly higher than those of the non-HE group in both lobar ICH and deep ICH derived from the training cohort (all *p* < 0.001), whereas there were no significant differences between Shape_MinorAxisLength_PHE (*p* = 0.390), Shape_VoxelVolume_PHE (*p* = 0.343), and Radscore_PHE (*p* = 0.327) between the HE and non-HE groups in patients with infratentorial (brain stem or cerebellum) ICH derived from the training cohort.

**Table 3:** Univariable analysis and correlation analysis for optimal PHE radiomics features and Radscore_HEA in the training cohort.

Variables	Shape_MinorAxisLength_PHE	Shape_VoxelVolume_PHE	Radscore_HEA
*Univariable analysis*			
Midline shift group (*n* = 85)	46.49 ± 11.19	25.83 ± 15.47	17.44 ± 20.09
No-midline shift group (n = 211)	32.47 ± 9.59	10.37 ± 8.09	−7.02 ± 14.86
*p*	< 0.001	< 0.001	< 0.001
Heterogeneity group (*n* = 96)	43.89 ± 10.96	21.63 ± 14.79	12.40 ± 19.98
Homogeneity group (*n* = 200)	32.94 ± 10.64	11.54 ± 10.26	−5.95 ± 16.89
*p*	< 0.001	< 0.001	< 0.001
Irregular shape group (*n* = 192)	40.17 ± 11.29	17.91 ± 13.57	5.88 ± 19.42
Regular shape group (*n* = 104)	29.71 ± 9.86	9.09 ± 8.77	−10.86 ± 15.76
*p*	< 0.001	< 0.001	< 0.001
*Correlation analysis*			
GCS score (*n* = 296)	*r* = −0.359	*r* = −0.326	*r* = −0.357
*p*	< 0.001	< 0.001	< 0.001
Onset_time (*n* = 296)	*r* = −0.248	*r* = −0.209	*r* = −0.234
*p*	< 0.001	< 0.001	< 0.001
Baseline hematoma volume (*n* = 296)	*r* = 0.909	*r* = 0.843	*r* = 0.905
*p*	< 0.001	< 0.001	< 0.001
Radscore_HEA (*n* = 296)	*r* = 0.858	*r* = 0.776	*r* = 0.850
*p*	< 0.001	< 0.001	< 0.001
Clinical score (*n* = 296)	*r* = 0.359	*r* = 0.315	*r* = 0.366
*p*	< 0.001	< 0.001	< 0.001

Data are noted as mean and standard deviation. PHE, perihematomal edema; GCS, Glasgow coma scale; HEA, hematoma.

### Multivariable analysis

Multivariable analysis showed that, in the training cohort, Radscore_PHE (odds ratio [OR] and 95% confidence interval [CI] 1.039 [1.003 - 1.077], *p* = 0.032) rather than Shape_MinorAxisLength_PHE (1.031 [0.974 - 1.092], *p* = 0.296) or Shape_VoxelVolume_PHE (1.034 [0.989 - 1.081], *p* = 0.142), was an independent risk factor for HE after adjustment for GCS score, Onset_time, baseline hematoma volume, hematoma shape, hematoma density, midline shift, and Radscore_HEA.

### Prediction performances

**Figure 4:**
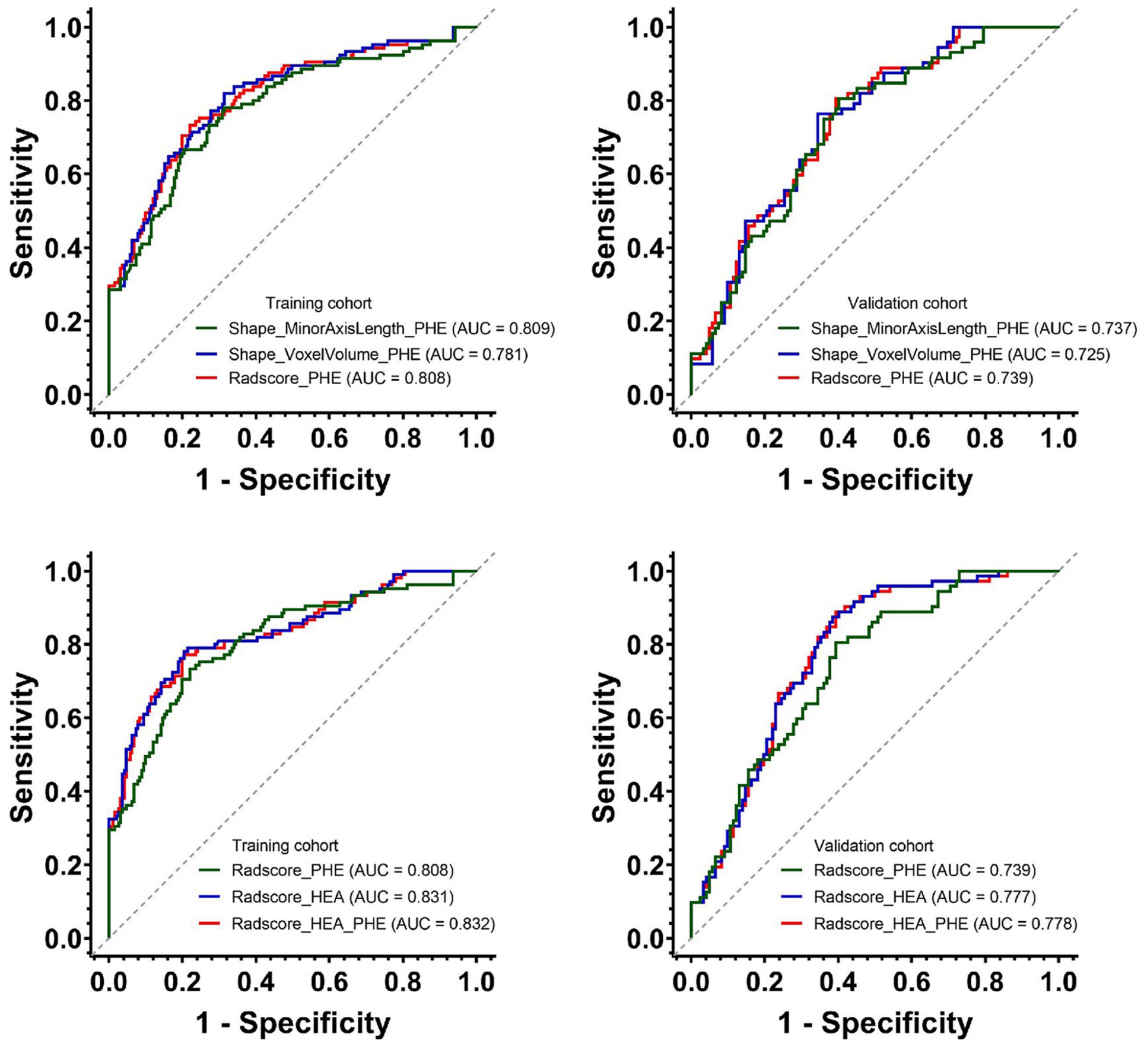
Predictive performances of Shape_MinorAxisLength_PHE, Shape_VoxelVolume_PHE and three Radscores for HE in the training and validation cohorts. PHE, perihematomal edema; HE, hematoma expansion.

The AUCs and 95% CIs of Shape_MinorAxisLength_PHE, Shape_VoxelVolume_PHE, and Radscore_PHE were 0.809 [0.756–0.862], 0.781 [0.725–0.838], and 0.808 [0.755–0.861] in the training cohort and 0.737 [0.668–0.807], 0.725 [0.654–0.796], and 0.739 [0.670–0.808] in the validation cohort, respectively (Figure 4). The AUCs and 95% CIs of Radscore_PHE, Radscore_HEA, and Radscore_HEA_PHE were 0.808 [0.759 – 0.851], 0831 [0.783 – 0.872], and 0.832 [0.785 – 0.873] in the training cohort and 0.739 [0.672 – 0.800], 0.777 [0.712 – 0.834], and 0.778 [0.713 – 0.834] in the validation cohort, respectively (Figure 4). No significant differences were found between the AUCs of these three Radscores both in the training and validation cohorts (Table 4).

**Table 4:** The Delong results of pairwise comparisons of the AUCs among three Radscores in the training and validation cohorts.

Delong test	Radscore_HEA	Radscore_PHE
*Training cohort*		
Radscore_PHE	*p* = 0.154	–
Radscore_HEA_PHE	*p* = 0.639	*p* = 0.068
*Validation cohort*		
Radscore_PHE	*p* = 0.089	–
Radscore_HEA_PHE	*p* = 0.596	*p* = 0.067

AUC, area under the curve; PHE, perihematomal edema; HEA, hematoma.

In addition, after incorporating Radscore_PHE, the predictive power of Radscore_HEA_PHE was slightly improved, although without statistical significance, in the training cohort, with an integrated discrimination improvement (IDI) and 95% CIs of (0.009 [−0.001 – 0.018], *p* = 0.086), whereas the predictive power of Radscore_HEA_PHE was slightly decreased with statistical significance in the validation cohort, with an IDI and 95% CIs of (−0.011 [−0.017 – −0.005], *p* < 0.001).

## Discussion

To the best of our knowledge, no previous study has investigated the relationship between PHE and HE using NCCT-based radiomics analysis. Our findings indicated that Shape_VoxelVolume_PHE, Shape_MinorAxisLength_PHE, and Radscore_PHE were associated with HE. Among these factors, only Radscore_PHE emerged as an independent predictor of HE. However, despite demonstrating predictive capability, the efficiency of Radscore_PHE appeared to be suboptimal, compared to that of Radscore_HEA, and failed to yield significant incremental value.

Currently, the pathophysiological mechanisms underlying the formation of HE and PHE are poorly understood. HE may indicate ongoing bleeding within the hematoma, secondary bleeding around the hematoma, or a combination of both (Schlunk and Greenberg, 2015). The formation of PHE is associated with clot retraction, inflammation, and erythrocyte lysis (Zheng et al., 2016). Several studies have suggested that PHE formation, inflammation, and HE are linked (Alvarez-Sabín et al., 2004; Silva et al., 2005; Florczak-Rzepka et al., 2012). However, studies predicting HE have predominantly focused on the characteristics of the hematoma itself (Morotti et al., 2020; Jiang et al., 2022), often overlooking the contributory role of PHE. A previous study suggested that reduced cerebral blood volume in the perihematomal region is associated with HE, suggesting a potential role for the perihematomal region in the pathophysiology of HE (Morotti et al., 2019b). Therefore, exploring the role of PHE in predicting HE is warranted.

Currently, the assessment of PHE has primarily been limited to volume-and density-related parameters (Selim and Norton, 2018; Huan et al., 2021), with studies investigating the association between PHE and HE focusing solely on absolute and relative PHE volume (Gebel et al., 2002; Rodriguez-Luna et al., 2016). Relying solely on the quantitative parameters of PHE volume may inadequately capture nuanced pathological changes. To address this gap, our study employed radiomics analysis to extract multidimensional information pertaining to PHE comprehensively. Remarkably, both Shape_VoxelVolume and Shape_MinorAxisLength of PHE were identified as optimal features associated with HE. Shape_MinorAxisLength represents the second largest axis length within the VOI. Our results showed that patients without HE had smaller PHE Shape_MinorAxisLength values, indicative of a more regular edema morphology. These findings not only reinforce the established relationship between PHE volume and HE (Rodriguez-Luna et al., 2016), but also identify the Shape_MinorAxisLength_PHE as a novel indicator associated with HE.

Although the PHE volume has been found to correlate with HE, it lacks independent predictive capability for HE (Rodriguez-Luna et al., 2016), in agreement with our findings. In this study, despite the associations of Shape_VoxelVolume_PHE, Shape_MinorAxisLength_PHE, Radscore_PHE, with HE, as well as their correlations with the aforementioned clinically relevant factors, imaging signs of ICH, and Radscore_HEA, only Radscore_PHE emerged as an independent risk factor for HE. Compared with PHE volume, Radscore_PHE contains more information about the PHE and may better describe its imaging characteristics. Thus, these findings not only provide a novel tool for predicting HE based on PHE characteristics, but also provide fresh evidence supporting the consideration of PHE treatment as a prospective therapeutic target for addressing HE in individuals with ICH.

To explore the predictive value of Radscore_PHE in HE, we developed a combined model integrating both Radscore_HEA and Radscore_PHE and subsequently assessed the predictive performance of the three Radscores for HE. Our investigation revealed that Radscore_PHE demonstrated predictive capability for HE, as evidenced by its comparable performance to Radscore_HEA, as indicated by slightly lower yet statistically insignificant differences in the AUC values across both training and validation cohorts. However, the results of the IDI test showed that Radscore_PHE did not offer a significant incremental predictive value for HE. This observation can be explained by the fact that the radiomics features of hematoma directly manifest as HE, whereas the radiomics features of PHE serve as an indirect representation of HE. Therefore, our results suggest that Radscore_PHE is a surrogate for Radscore_HEA although it can independently predict HE.

This study had several limitations. First, it was retrospective in nature, potentially leading to an incomplete consideration of the influencing factors, such as length of untreated hypertension during hospitalization or presence of anticoagulation/antiplatelet therapy. The relatively small sample size may have introduced selection bias. Second, our study only included patients with ICH within 6 h of ICH onset. Future investigations should validate our findings in patients outside the 6-h window. Third, while efforts were made to achieve consensus among the raters regarding the segmentation of the VOI for hematoma and PHE, the influence of other similar densities on image segmentation cannot be completely ruled out. Finally, although Radscore_PHE independently predicted HE, further elucidation of its biological basis is necessary to develop more effective therapeutic strategies against HE after ICH. In addition, previous reports have indicated that enhancing the classification accuracy and prediction significance scores may be achieved by incorporating additional clinical scores and utilizing a composite of multiple classifiers and prediction models (Zhou, 2017). This approach may also contribute to the refinement of the Radscore_PHE.

In conclusion, our study demonstrated that Radscore_PHE, based on Shape_VoxelVolume and Shape_MinorAxisLength of PHE, could independently predict HE, but it is a surrogate for Radscore_HEA with no significant incremental value to Radscore_HEA. Future studies involving longitudinal follow-up to implement and validate other imaging modalities, such as MRI, may provide valuable insights into PHE and its relationship with HE.

## Data availability statement

The original contributions presented in the study are included in the article/Supplementary material, further inquiries can be directed to the corresponding author.

## Ethics statement

The studies involving humans were approved by The Ethics Committee of the Second Affiliated Hospital of Chongqing Medical University. The studies were conducted in accordance with the local legislation and institutional requirements. The ethics committee/institutional review board waived the requirement of written informed consent for participation from the participants or the participants’ legal guardians/next of kin because the retrospective nature of the study.

## Author contributions

ZZ: Conceptualization, Data curation, Formal analysis, Funding acquisition, Investigation, Methodology, Writing – original draft, Writing – review & editing. XW: Formal analysis, Methodology, Writing – review & editing. YC: Data curation, Writing – review & editing. YT: Data curation, Writing – review & editing. YZ: Data curation, Writing – review & editing. TH: Data curation, Writing – review & editing. HZ: Data curation, Writing – review & editing. QL: Formal Analysis, Methodology, Writing – review & editing. DG: Conceptualization, Funding acquisition, Methodology, Project administration, Supervision, Writing – review & editing.

## Footnotes

^1^ https://en.shukun.net/scientific.html

^2^ http://www.itksnap.org/pm.s/pm.com/.php

^3^ https://www.python.org/

^4^ https://www.r-project.org/

## References

[ref1] Alvarez-Sabín J.´. Delgado P. Abilleira S. Molina C. Arenillas J. Ribó M.. (2004). Temporal profile of matrix metalloproteinases and their inhibitors after spontaneous intracerebral hemorrhage: relationship to clinical and radiological outcome. Stroke 35, 1316–1322. doi: 10.1161/01.str.0000126827.69286.90, PMID: 15087562

[ref2] An S. Kim T. Yoon B. (2017). Epidemiology, risk factors, and clinical features of intracerebral hemorrhage: An update. J. Stroke 19, 3–10. doi: 10.5853/jos.2016.00864, PMID: 28178408 PMC5307940

[ref3] Arima H. Wang J. G. Huang Y. Heeley E. Skulina C. Parsons M. W.. (2009). Significance of perihematomal edema in acute intracerebral hemorrhage: the INTERACT trial. Neurology 73, 1963–1968. doi: 10.1212/WNL.0b013e3181c55ed3, PMID: 19996072 PMC2796457

[ref4] Barras C. D. Tress B. M. Christensen S. Mac Gregor L. Collins M. Desmond P. M.. (2009). Density and shape as CT predictors of intracerebral hemorrhage growth. Stroke 40, 1325–1331. doi: 10.1161/strokeaha.108.536888, PMID: 19286590

[ref5] Brouwers H. B. Greenberg S. M. (2013). Hematoma expansion following acute intracerebral hemorrhage. Cerebrovasc. Dis. 35, 195–201. doi: 10.1159/000346599, PMID: 23466430 PMC3743539

[ref6] Demchuk A. Dowlatshahi D. Rodriguez-Luna D. Molina C. Blas Y. Dzialowski I.. (2012). Prediction of haematoma growth and outcome in patients with intracerebral haemorrhage using the CT-angiography spot sign (PREDICT): a prospective observational study. Lancet Neurol. 11, 307–314. doi: 10.1016/s1474-4422(12)70038-8, PMID: 22405630

[ref7] Florczak-Rzepka M. Grond-Ginsbach C. Montaner J. Steiner T. (2012). Matrix metalloproteinases in human spontaneous intracerebral hemorrhage: an update. Cerebrovasc. Dis. 34, 249–262. doi: 10.1159/000341686, PMID: 23052179

[ref8] Gebel J. Jauch E. Brott T. Khoury J. Sauerbeck L. Salisbury S.. (2002). Natural history of perihematomal edema in patients with hyperacute spontaneous intracerebral hemorrhage. Stroke 33, 2631–2635. doi: 10.1161/01.str.0000035284.12699.84, PMID: 12411653

[ref9] Huan R. Li Y. Tan J. Tang J. Huang N. Cheng Y. (2021). The Hounsfield unit of Perihematomal edema is associated with poor clinical outcomes in intracerebral hemorrhage. World Neurosurg. 146, e829–e836. doi: 10.1016/j.wneu.2020.11.025, PMID: 33189917

[ref10] Huang X. Wang D. Ma Y. Zhang Q. Ren J. Zhao H.. (2023). Perihematomal edema-based CT-radiomics model to predict functional outcome in patients with intracerebral hemorrhage. Diagn. Interv. Imaging 104, 391–400. doi: 10.1016/j.diii.2023.04.008, PMID: 37179244

[ref11] Huang X. Wang D. Zhang Q. Ma Y. Zhao H. Li S.. (2022). Radiomics for prediction of intracerebral hemorrhage outcomes: a retrospective multicenter study. Neuro Image. Clin. 36:103242. doi: 10.1016/j.nicl.2022.103242, PMID: 36279754 PMC9668657

[ref12] Jiang Y. Xu X. Wang R. Chen C. (2022). Efficacy of non-enhanced computer tomography-based radiomics for predicting hematoma expansion: a meta-analysis. Front. Oncol. 12:973104. doi: 10.3389/fonc.2022.973104, PMID: 36703784 PMC9872032

[ref13] Morotti A. Arba F. (2020). A novel 10-point score system to predict early hematoma growth in patients with spontaneous intracerebral hemorrhage. Neurology 10, 632–643. doi: 10.3389/fneur.2019.01417, PMID: 32116989 PMC7018853

[ref14] Morotti A. Arba F. Boulouis G. Charidimou A. (2020). Noncontrast CT markers of intracerebral hemorrhage expansion and poor outcome: a meta-analysis. Neurology 95, 632–643. doi: 10.1212/wnl.0000000000010660, PMID: 32847959

[ref15] Morotti A. Boulouis G. Dowlatshahi D. Li Q. Barras C. Delcourt C.. (2019a). Standards for detecting, interpreting, and reporting noncontrast computed tomographic markers of intracerebral hemorrhage expansion. Ann. Neurol. 86, 480–492. doi: 10.1002/ana.25563, PMID: 31364773

[ref16] Morotti A. Busto G. Bernardoni A. Tamborino C. Fainardi E. (2019b). Association between perihematomal cerebral blood volume and intracerebral hemorrhage expansion: a computed tomography perfusion study. Ann. Neurol. 85, 943–947. doi: 10.1002/ana.25466, PMID: 30864197

[ref17] Rodriguez-Luna D. Stewart T. Dowlatshahi D. Kosior J. Aviv R. Molina C.. (2016). Perihematomal edema is greater in the presence of a spot sign but does not predict intracerebral hematoma expansion. Stroke 47, 350–355. doi: 10.1161/strokeaha.115.011295, PMID: 26696644

[ref18] Schlunk F. Greenberg S. M. (2015). The pathophysiology of intracerebral hemorrhage formation and expansion. Transl. Stroke Res. 6, 257–263. doi: 10.1007/s12975-015-0410-1 26073700

[ref19] Selim M. Norton C. (2018). Perihematomal edema: implications for intracerebral hemorrhage research and therapeutic advances. J. Neurosci. Res. 98, 212–218. doi: 10.1002/jnr.24372, PMID: 30575082 PMC6588515

[ref20] Silva Y. Leira R. Tejada J. Lainez J. Castillo J. Dávalos A. (2005). Molecular signatures of vascular injury are associated with early growth of intracerebral hemorrhage. Stroke 36, 86–91. doi: 10.1161/01.STR.0000149615.51204.0b, PMID: 15550687

[ref21] Staykov D. Wagner I. Volbers B. Hauer E. Doerfler A. Schwab S.. (2011). Natural course of perihemorrhagic edema after intracerebral hemorrhage. Stroke 42, 2625–2629. doi: 10.1161/strokeaha.111.618611, PMID: 21737803

[ref22] Steiner T. Bosel J. (2010). Options to restrict hematoma expansion after spontaneous intracerebral hemorrhage. Stroke 41, 402–409. doi: 10.1161/STROKEAHA.109.552919, PMID: 20044536

[ref23] Venkatasubramanian C. Mlynash M. Finley-Caulfield A. Eyngorn I. Kalimuthu R. Snider R. W.. (2011). Natural history of perihematomal edema after intracerebral hemorrhage measured by serial magnetic resonance imaging. Stroke 42, 73–80. doi: 10.1161/STROKEAHA.110.590646, PMID: 21164136 PMC3074599

[ref24] Wada A. Fox S. Gladstone T.. (2007). CT angiography "spot sign" predicts hematoma expansion in acute intracerebral hemorrhage. Stroke 38, 1257–1262. doi: 10.1161/01.STR.0000259633.59404.f3 17322083

[ref25] Yang W. S. Li Q. Li R. Liu Q. J. Wang X. C. Zhao L. B.. (2018). Defining the optimal midline shift threshold to predict poor outcome in patients with Supratentorial spontaneous intracerebral hemorrhage. Neurocrit. Care. 28, 314–321. doi: 10.1007/s12028-017-0483-7, PMID: 29139015

[ref26] Zheng H. Chen C. Zhang J. Hu Z. (2016). Mechanism and therapy of brain edema after intracerebral hemorrhage. Cerebrovasc. Dis. 42, 155–169. doi: 10.1159/000445170 27110940

[ref27] Zhou Y. (2017). Neuroimaging in mild traumatic brain injury. London, United Kingdom: Nova Science Publisher.

[ref28] Zhou Z. Zhou H. Song Z. Chen Y. Guo D. Cai J. (2021). Location-specific Radiomics score: novel imaging marker for predicting poor outcome of deep and lobar spontaneous intracerebral hemorrhage. Front. Neurosci. 15:766228. doi: 10.3389/fnins.2021.766228, PMID: 34899168 PMC8656420

