# Supplementary material for: The relationship between perihematomal edema and hematoma expansion in acute spontaneous intracerebral hemorrhage: an exploratory radiomics analysis study
Source: Front Neurosci. 2024 Apr 30;18:1394795. doi: 10.3389/fnins.2024.1394795 (PMC11091303; doi:10.3389/fnins.2024.1394795)
Supplement: Supplementary file 1 [file Data_Sheet_1.docx]

**SUPPLEMENTAL MATERIALS**

**MATERIALS AND METHODS**

**Radiomics features extraction and selection**

Inter-observer and intra-observer reproducibility analyses was performed on 50 patients randomly selected from the training cohort.

The volumes of interests (VOIs) of hematoma and perihematomal edema (PHE) were manually segmented again by the same neuroradiologist at initial time point and after 2 weeks interval, as well as by another trained neuroradiologist using the same method. Totally 107 radiomics features derived from 8 feature groups including neighborhood gray level dependency matrix, neighborhood gray difference matrix, gray level run length matrix, gray level size zone matrix, gray level co-occurrence matrix, 2D shape, 3D shape, and first-order statistics were extracted from these VOIs using the Python software. The interclass correlation coefficient (ICC) of between those radiomics features was calculated to determine inter-observer and intra-observer reliabilities.

**RESULTS**

**Features extraction and selection**

The groups and corresponding numbers of the radiomics features are shown in Figure S1. During features selection of radiomics features of PHE, 37 of the 107 radiomics features were retained after the *t*-test, with a significance level of *P* < 0.05. Subsequently, 2 radiomics features were obtained after elastic net regression (with parameters of L1_ratio = 0.5, cv = 10, max_iter = 10000) and alpha value of 0.097, (Figure S1). During features selection of radiomics features of hematoma, 33 of the 107 radiomics features were retained after the *t*-test (with a significance level of *P* < 0.05), and 8 radiomics features were obtained after elastic net regression (with parameters of L1_ratio = 0.5, cv = 10, max_iter = 10000) and alpha value of 0.047 (Figure S1).


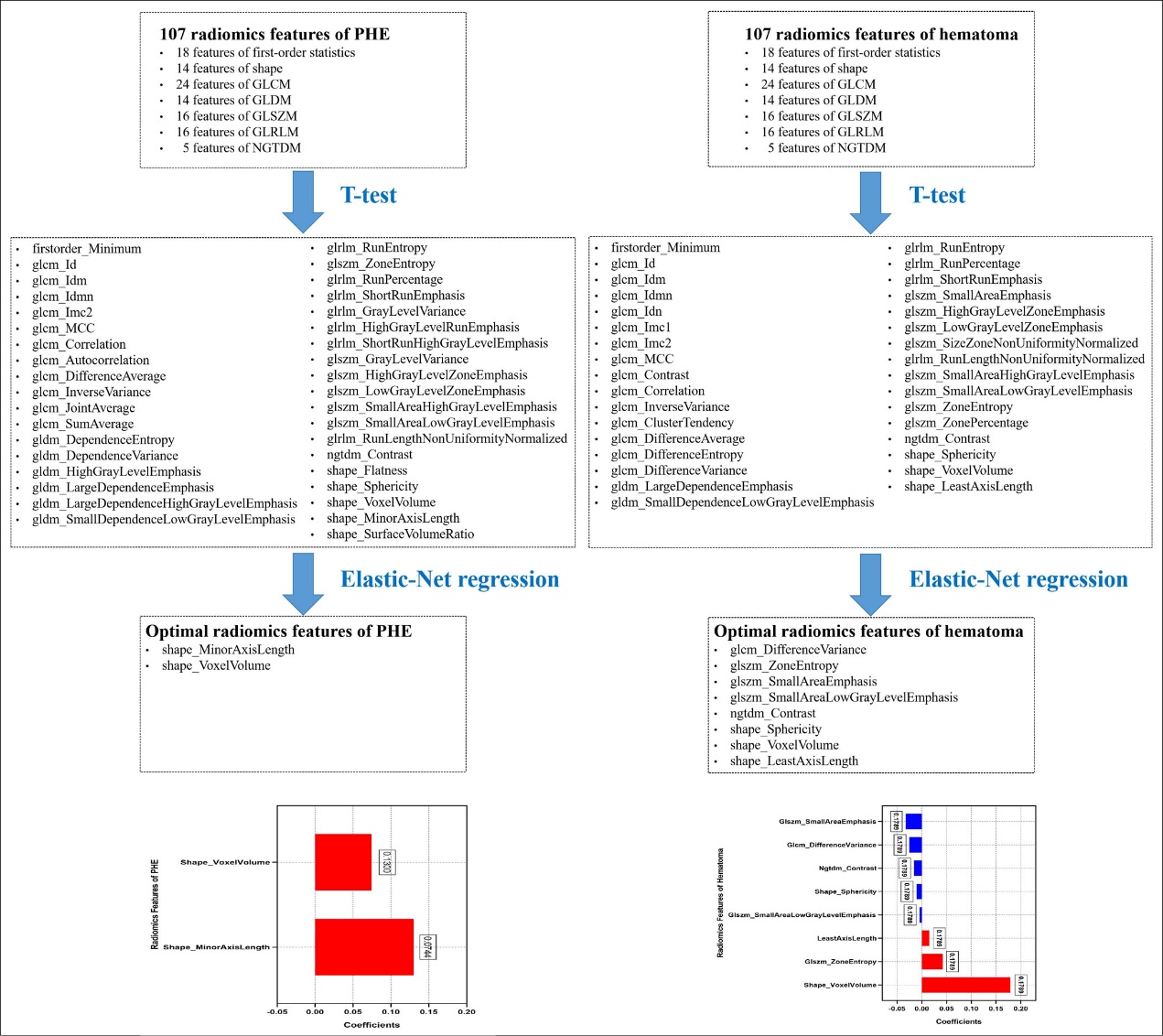


**Figure S1 |** Features extraction and selection of radiomics features of hematoma and PHE. PHE, perihematomal edema.
